# Biosynthesis, regulation, and engineering of microbially produced branched biofuels

**DOI:** 10.1186/s13068-019-1424-9

**Published:** 2019-04-13

**Authors:** Wenqin Bai, Weitao Geng, Shaojie Wang, Fuzhong Zhang

**Affiliations:** 1 Department of Energy, Environmental and Chemical Engineering, Washington University in St. Louis, Saint Louis, MO 63130 USA; 2 Division of Biological & Biomedical Sciences, Washington University in St. Louis, Saint Louis, MO 63130 USA; 3 Institute of Materials Science & Engineering, Washington University in St. Louis, Saint Louis, MO 63130 USA

**Keywords:** Advanced biofuels, Branched fuels, Branched fatty acids, Branched alcohols, Cyclopropane fatty acid

## Abstract

The steadily increasing demand on transportation fuels calls for renewable fuel replacements. This has attracted a growing amount of research to develop advanced biofuels that have similar physical, chemical, and combustion properties with petroleum-derived fossil fuels. Early generations of biofuels, such as ethanol, butanol, and straight-chain fatty acid-derived esters or hydrocarbons suffer from various undesirable properties and can only be blended in limited amounts. Recent research has shifted to the production of branched-chain biofuels that, compared to straight-chain fuels, have higher octane values, better cold flow, and lower cloud points, making them more suitable for existing engines, particularly for diesel and jet engines. This review focuses on several types of branched-chain biofuels and their immediate precursors, including branched short-chain (C4–C8) and long-chain (C15–C19)-alcohols, alkanes, and esters. We discuss their biosynthesis, regulation, and recent efforts in their overproduction by engineered microbes.

## Introduction

High petroleum prices and increasing concerns over energy security and climate change are driving the development of renewable biofuels in recent years [1, 2]. Bioethanol has been commercially used as a gasoline replacement in major markets of the world. However, this early generation of biofuel has several problems, such as low energy density and high hygroscopicity, leading to storage and transportation problems [3]. Recent development in microbial engineering has enabled the bioproduction of a suite of biofuel molecules, such as 1-butanol, isobutanol, limonene, hydrogenated farnesene, and fatty acid-derived alkanes, alkenes, alcohols, and esters [4–11]. Based on the chain length or the number of carbon atoms in their molecules, biofuels can be divided into short-chain (C4-C8), medium-chain (C9-C14), and long-chain (C15–C20) fuels. Based on their chain structures, biofuels can be divided into straight-chain or branched-chain biofuels. Compared to straight-chain biofuels, their branched-chain counterparts often have better physical and combustion properties. Branched short-chain alcohols such as isobutanol and 3-methyl-1-butanol have higher octane values than their linear-chain counterparts [3, 12]. Branched long-chain fuels offer improved properties such as lower freezing point, better cold flow, and lower cloud point, compared with their straight-chain counterparts [13, 14], which are vital to practical biofuel use at low temperature and high altitude, particularly for jet fuels.

While production of straight-chain biofuels has been extensively reviewed [15, 16], this review targets recent progress in the microbial synthesis and overproduction of branched biofuels and their immediate precursors. Current branched fuels mostly contain only methyl branches. Ethyl and higher branched structures are relatively rare in nature and have not been extensively engineered for energy applications. Branched short-chain alcohols and esters, such as isopentenol, isobutanol, 2-methyl-1-butanol, and 3-methyl-1-butanol, are mostly derived from branched α-keto acids. Branched long-chain fuels are mostly derived from lipid fatty acids. These two types of biofuels are the major focus of this review. Branched medium-chain biofuels can be derived by engineering the isoprenoid pathway, which has been excellently reviewed elsewhere [16, 17] thus will not be discussed here. Medium-chain biofuels can also be biosynthesized by truncating the intermediates during lipid fatty acid biosynthesis [18, 19], which will be discussed together with short- and long-chain fuels. Among the branched long-chain fuels, the position of the branch can be either at the terminus or the middle of the chain. These two types of compounds were synthesized by different routes of the lipid metabolic pathway and thus are discussed separately. For each pathway, we briefly discuss the natural function of the branched compounds that lead to the synthesis of biofuels, followed by an introduction of the pathway, its regulation, and recent engineering efforts in the overproduction of branched biofuels. General metabolic engineering strategies to improve titers, yields, and productivities have been reviewed elsewhere and will not be discussed [20–23]. Many of the advanced biofuels are in their early developmental stage; therefore, their scalable production and real-world application will not be the focus of this review.

## Branched short-chain biofuels

In recent years, considerable advances have been achieved by engineering microorganisms to produce branched short-chain (C4–C8) alcohols and esters. These compounds were originally identified as flavor compounds in the food industry [24] and are derived from branched-chain α-keto acids or amino acids via a pathway proposed by Ehrlich [25]. Because of the broad substrate range of the enzymes in the last two steps of the Ehrlich pathway, the pathway becomes the basis for production of a wide range of branched short-chain compounds, including branched biofuels [3]. Key genes in the Ehrlich pathway together with genes in the biosynthesis of α-keto acids have been engineered to improve titers and yields of branched short-chain compounds in various microorganisms, such as *Escherichia coli* [3], *Bacillus subtilis* [26], *Saccharomyces cerevisiae* [27] and *Ralstonia eutropha* [28]. Similar to straight short-chain alcohols, branched short-chain alcohols interfere with cell membrane’s function as a barrier, thus usually exhibiting cellular toxicity when produced in microbial hosts [29].

### Biosynthesis of branched short-chain alcohols and esters

**Fig. 1:**
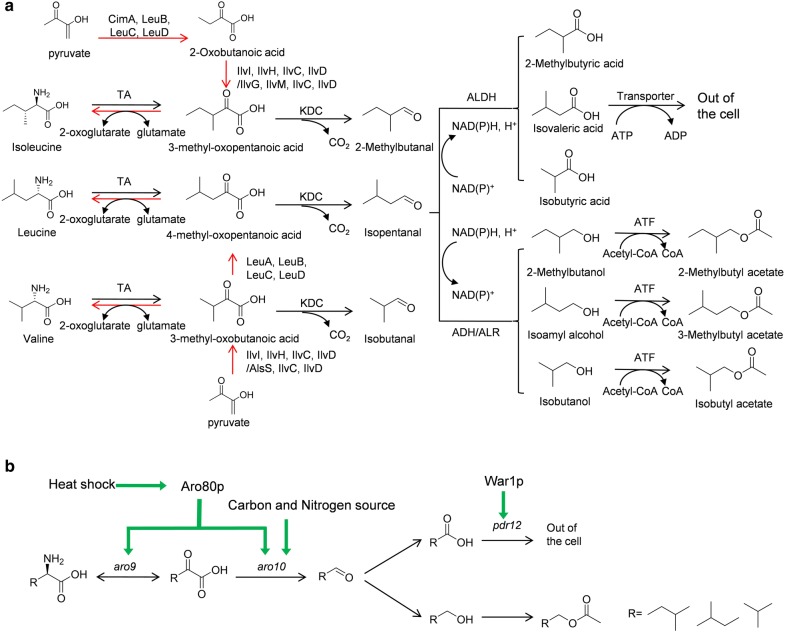
Biosynthetic pathways of branched short-chain alcohols and esters and their regulation in *S. cerevisiae*. **a** Biosynthesis of branched-chain amino acids from pryurate is shown by red arrows. IlvI: acetolactate synthase large subunit; IlvH: acetolactate synthase small subunit; IlvC: 2-hydroxy-3-ketol-acid reductoisomerase; IlvD: dihydroxy-acid hydratase; AlsS: acetolactate synthase; IlvG: acetolactate synthase II large subunit; IlvM: acetolactate synthase II large subunit; LeuA: 2-isopropylmalate synthase; LeuB: 3-propylmalate dehydrogenase; LeuC/D: isopropylmalate isomerase; CimA: citramalate synthase. The Ehrlich pathway in *S. cerevisiae* is shown in black arrows. TA: transaminase; KDC: α-ketoacid decarboxylase; ADH: alcohol dehydrogenases; ALDH: aldehyde dehydrogenase; ALR: aldehyde reductase; ATF: alcohol acetyltransferases. **b** A schematic overview of the regulation of branched short-chain alcohols and esters biosynthesis in *S. cerevisiae*

In the Ehrlich pathway (Fig. 1a), branched-chain amino acids (valine, leucine, and isoleucine) serve as the precursors and are first converted to the corresponding α-keto acids (3-methyl-oxopentanoic acid, 4-methyl-oxopentanoic acid and 3-methyl-oxobutyric acid) by transaminase (TA). Branched-chain α-keto acids can be also synthesized from carbohydrate feedstock, such as glucose, via pyruvate. Subsequently, these α-keto acids are decarboxylated by α-keto acid decarboxylase (KDC) to form the corresponding aldehydes (2-methylbutanal, isopentanal, and isobutanal). Finally, these aldehydes are oxidized by aldehyde dehydrogenase (ALDH) to branched short-chain fatty acids (2-methylbutyric acid, isovaleric acid, and isobutyric acid) or reduced by alcohol dehydrogenases (ADH) or aldehyde reductase (ALR) to branched short-chain alcohols (2-methylbutanol, isoamyl alcohol, and isobutanol). The alcohols can be combined with acyl-CoAs by alcohol acetyltransferases (ATFs) to form various esters.

### Regulation of the Ehrlich pathway in *S. cerevisiae*

Both pathway-specific activators and general regulatory mechanisms participate in the regulation of higher alcohol production in the context of the Ehrlich pathway (Fig. 1b).

Iaqui et al. [30] first identified Aro80p as a pathway-specific transcriptional activator involved in the induction of ARO9 transaminase and ARO10 α-keto acid decarboxylase genes in the presence of the aromatic amino acid tryptophan, phenylalanine, and tyrosine. A WRCCGWSATTTRCCG motif in the *aro10* and *aro9* promoters was needed for the binding of Aro80p [31]. Then, Lee et al. [32] found that *aro9* and *aro10* transcription also requires the GATA activators (Gat1 and Gln3), which mediate the nitrogen catabolite repression by activating GATA genes in low nitrogen conditions. The result shows that Aro80p not only induces its target genes *aro10* and *aro9* by binding to their promoters, but also by the recruitment of the GATA activators. In addition, another activator War1p was identified to induce the transcription of the transporter Pdr12 encoding gene *pdr12*. The activator has a cis-acting element in the promoter of *pdr12* and becomes active upon phosphorylation in response to the nonphysiological substrates benzoate and sorbate [33].

In addition, the culture conditions would also regulate the Ehrlich pathway. Lee et al. [34] found that the heat shock stress was able to affect *aro10* and *aro9* transcription. The expression level of the *aro10* and *aro9* genes in an *aro80* knockdown strain suggests that their transcription is activated by Aro80 under heat shock stress in *S. cerevisiae*. Furthermore, the Ehrlich pathway is also regulated in a carbon and nitrogen source-dependent manner. For example, constitutive overexpression of the ARO10 decarboxylase gene does not increase the 3-phenylpyruvate decarboxylase activity during growth on a medium supplemented with glucose and ammonium sulfate. However, the replacement of either the ammonium sulfate with phenylalanine or glucose with ethanol results in an obvious increase of 3-phenylpyruvate decarboxylase activity [35].

### Metabolic engineering for the production of branched short-chain alcohols and esters

Current overproduction of branched short-chain biofuels is mainly focused on the optimization and/or modification of the Ehrlich pathways, the improvement of precursor pool, and the optimization of cofactor availability. *S. cerevisiae* has the natural ability to produce fatty alcohols. However, titers of branched short-chain biofuel in wild-type *S. cerevisiae* are very low [25, 36]. To enhance the flux through the Ehrlich pathway, Kondo et al. [26] overexpressed α-keto acid decarboxylase (KDC) from *Lactococcus lactis* and an alcohol dehydrogenase Adh6p from *S. cerevisiae* (Fig. 1a). Subsequently, the acetolactate synthase Ilv2, which catalyzes the first step of the valine synthetic pathway, was overexpressed to enhance the flux to 2-keto-3-methylvalerate. Meanwhile, the pyruvate decarboxylase PDC1 was eliminated to reduce the flux from pyruvate to ethanol. The resulting strain produced isobutanol at a titer of 143 mg/L, which was 13-fold higher than that of the wild-type strain. Built upon this work, Matsuda et al. [37] further eliminated a competing pathway by deletion of the pyruvate dehydrogenase complex LPD1 and enhanced the cellular NADPH content by overexpression of the malate dehydrogenase MAE1, reaching an isobutanol titer of 1.62 g/L.

Besides producing branched short-chain alcohols and esters from their natural pathways in *S. cerevisiae,* the Ehrlich pathway has also been engineered in a series of heterologous hosts that lack the Ehrlich pathway but offer other fermentative advantages. These heterologous hosts include *E. coli*, *B. subtilis*, *Corynebacterium glutamicum*, *Brevibacterium flavum R. eutropha*, and *Synechococcus elongatus* 7942. One common strategy is reconstruction of the Ehrlich pathway in a heterologous host via combinatorial gene overexpression and optimization by deletion of key competing genes. For example, Atsumi et al. [3] constructed the Ehrlich pathway by expressing one of the five α-keto acid decarboxylases (Pdc6p, Aro10p and Thi3p from *S. cerevisiae*, KIVD from *L. lactics* and PDC from *Clostridium acetobutylicum*) along with an alcohol dehydrogenases Adh2 from *S. cerevisiae* to produce isobutanol in *E. coli*. The engineered Ehrlich pathway uses endogenous α-keto acids as precursors in *E. coli*, whose cellular pool was enhanced by deleting multiple competing pathways/enzymes, including an aldehyde-alcohol dehydrogenase (encoded by *adhE*), a lactate dehydrogenase A (encoded by *ldhA*), a fumarate reductase (encoded by *frdAB*), a regulator of fumarate and nitrate reduction (encoded by *fnr*), and a phosphate acetyltransferase (encoded by *pta*) that contributes to by-product formation. In combination with the overexpression of the *ilvIHCD* genes for 2-ketoisovalerate biosynthesis, isobutanol was produced at a titer of 22 g/L. Similarly, 2.62 g/L of isobutanol was produced in *B. subtilis* by engineering an Ehrlich pathway together with the overexpression of the acetolactate synthase genes (*alsS*, *ilvC*, and *ilvD* as shown in Fig. 1) responsible for the synthesis of 2-ketoisovalerate [27]. *C. glutamicum*, the most widely used branched-chain amino acid producer in fermentation industry, has a natural ability to accumulate 2-ketoisovalerate and 2-keto-3-methylvalerate at high intracellular concentrations [38]. By overexpressing an α-keto acid decarboxylase (encoded by *aro10* from *S. cerevisiae*) and an alcohol dehydrogenase (encoded by *yqhD* from *E. coli*) and reducing the activity of the branched-chain amino acid transaminase, 3-methyl-1-butanol was produced at 2.76 g/L [38]. The Ehrlich pathway has also been engineered in *R. eutropha* [28], leading to the production of 3-methyl-1-butanol and isobutanol at 140 mg/L using CO_2_ as the carbon source and electricity as reducing power, and in *B. flavum* [39], leading to the production of isobutanol, 2-methyl-1-butanol, and 3-methyl-1-butanol using duckweed as feedstock. In addition, branched short-chain alcohols can be produced in engineered cyanobacteria using CO_2_ as the carbon source and sunlight energy. In *S. elongatus* 7942, a 5-step heterologous biosynthetic pathway was engineered, leading to the production of isobutanol at 450 mg/L [40]. Table 1 summarizes the theoretical yields of the above-mentioned biofuels, and their reported yields and titers in different hosts.

**Table 1:** Theoretical yields and achieved yields and titers of the branched biofuels in different hosts

Product	Theoretical yields (g/g glucose)	Achieved yields (g/g glucose) and titer (g/L) in different hosts													
		*E. coli*		*S. cerevisiae*		*B. subtilis*		*C. glutamicum*		*B. flavum*		*R. eutropha*		*S. elongatus*	
		Yield	Titer	Yield	Titer	Yield	Titer	Yield	Titer	Yield	Titer	Yield	Titer	Yield	Titer
Isobutanol	0.42	0.42 [98]	50 [99]	0.016 [37]	1.6 [37]	0.066 [27]	2.6 [27]	0.32 [100]	72.69 [100]	0.089 [39]	5.36 [39]	–	0.85 [28]	–	0.45 [40]
3-methyl-1-butanol	0.33	0.11 [4]	9.5 [4]	0.0076 [101]	0.77 [101]	–	Trace [27]	0.10 [38]	2.8 [38]	0.013 [39]	0.79 [39]	–	0.57 [28]	–	–
2-methyl-1-butanol	0.38	0.17 [102]	1.25 [102]	0.0045 [103]	0.18 [103]	–	Trace [27]	0.02 [38]	0.37 [38]	0.032 [39]	1.95 [39]	–	–	–	–
C_17:0 (1)_^a^	0.34	0.010 [59]	0.21 [59]	–	–	–	–	–	–	–	–	–	–	–	–

^a^Terminally branched LCFAs were always produced as mixtures, here the theoretical yield was calculated assuming a single product of C_17:0 (1)_

In addition, by overexpressing the endogenous alcohol acyltransferase (encoded by *atf1*) that catalyzes the condensation step of branched-chain alcohols with acetyl-CoA [41], isobutanol, 3-methyl-1-butanol, and 2-methyl-1-butanol were converted to isobutyl acetate, 3-methyl-1-butylacetate, and 2-methyl-1-butyl acetate with a titer of 260.2 mg/L, 296.1 mg/L, and 289.6 mg/L, respectively. For the biosynthesis of branched short-chain esters, Rodriguez et al. [42] engineered *E. coli* using the alcohol *O*-acyltransferase (ATF) from *S. cerevisiae*. By combining different acyl-CoA molecules found in nature with various alcohol biosynthetic pathways, a multitude of esters were obtained. In particular, they achieved high-level production of isobutyl acetate from glucose (17.2 g/L). Wax ester synthase/acyl-coenzyme A: diacylglycerol acyltransferase (WS/DGAT), which catalyzes the esterification of fatty acyl-CoAs and short-chain alcohols, was also introduced into *E. coli* for the biosynthesis of fatty acid short-chain esters (FASE). A titer of 209 ± 2.6 mg/L FASEs, 50% of which being fatty acid branched-chain esters (FABCEs), was obtained [43].

## Terminally branched long-chain fuels

Long-chain fatty acids (LCFAs) in the range of C14–C20 are the major membrane component for almost all organisms except for a few archaea species, which have isoprenoid-derived membranes [44]. While the most common LCFAs have straight chains, some Gram-positive organisms, such as the *Bacilli*, *Staphylococci*, and *Streptomycetes*, produce terminally methyl-branched LCFAs as the acyl constituents of membrane lipids. The methyl branch is usually found at either the ω-2 or ω-3 position of the acyl group and is called iso- or anteiso-branched fatty acids, respectively [45]. These terminally branched LCFAs are believed to increase the fluidity and lower the phase transition temperature of their lipid components [46]. Similarly, branched LCFA-derived biofuels have significantly lower melting temperatures than their straight-chain counterparts, offering improved cold flow properties [13].

### Biosynthesis of terminally branched long-chain fatty acids

**Fig. 2:**
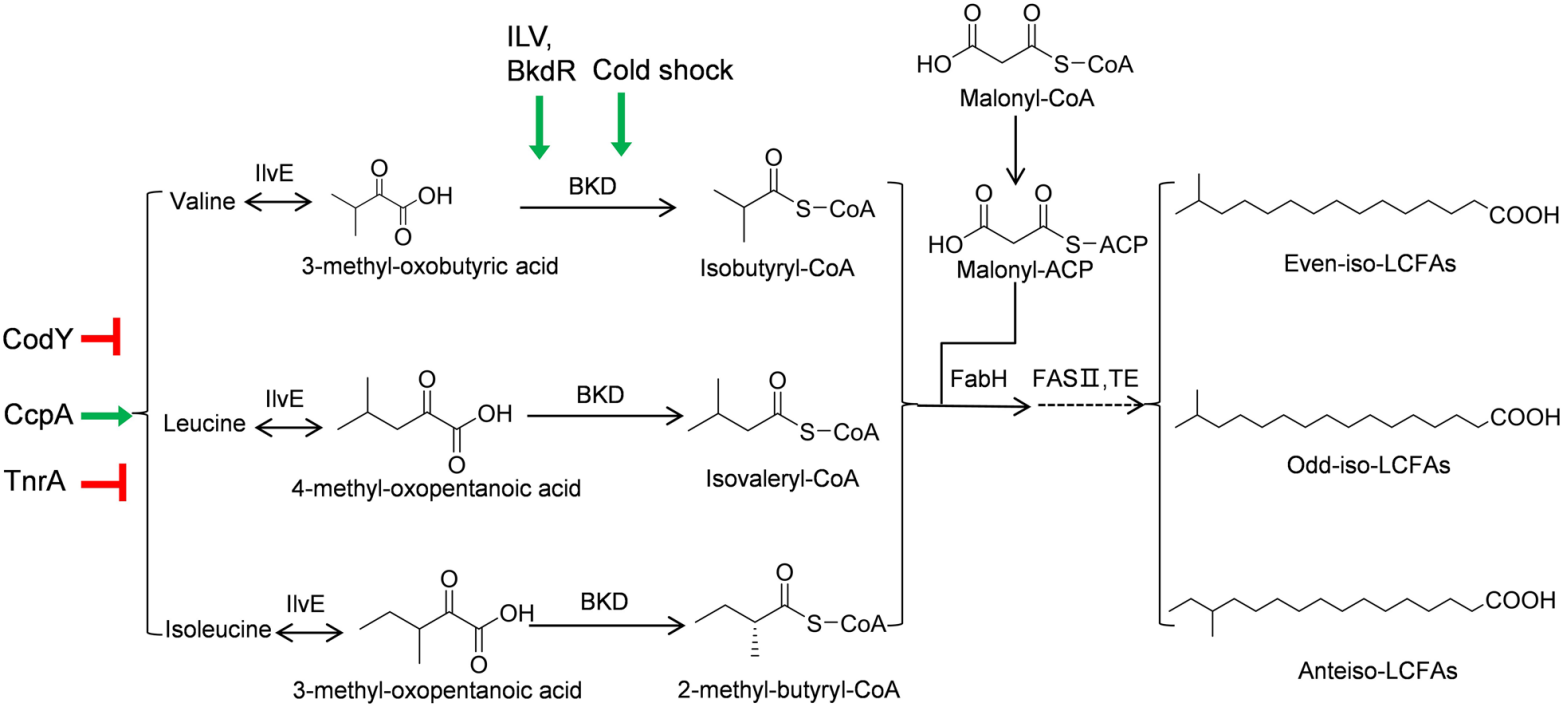
Terminally branched long-chain fatty acid biosynthetic pathways and its regulation in *B. subtilis*. IlvE: branched-chain aminotransferase; BKD: branched-chain α-keto acid dehydrogenase: BKD is composed of two E1 subunits (E1α: dehydrogenase; E1β: decarboxylase, encoded by *bkdAA* and *bkdAB*), one E2 subunit (lipoamide acyltransferase, encoded by *bkdB*), and one E3 subunit (dihydrolipoamide dehydrogenase, encodedby *lpdV*); FabH: β-ketoacyl-acyl carrier protein synthase III; FASII: type II fatty acid synthase (FabG, FabA/FabZ, FabI, and FabB/FabF); TE: thioesterase. Repression is show by red lines, and activation is shown by a green arrow

The biosynthetic pathway of terminally branched LCFAs in *B. subtilis* is well characterized [47–49] as shown in Fig. 2. This pathway used branched-chain α-keto acids as the precursors [49], which are first activated to short-chain acyl-CoAs by branched-chain α-keto acid dehydrogenases (BKD, encoded by the *bkd* operon). C4 and C5 branched-chain acyl-CoAs are the primer for the biosynthesis of terminally branched LCFAs, and malonyl-CoA is the chain extender of the primer. β-ketoacyl-acyl carrier protein synthase III (FabH) catalyzes the first condensation step by condensing branched-chain acyl-CoAs with malonyl-acyl carrier protein (malonyl-ACP). The resulting β-ketoacyl-ACP is then elongated through repeated cycles of reactions catalyzed by the multienzyme fatty acid type II biosynthesis system (FASII) to yield terminally branched long-chain acyl-ACPs. Thioesterase can hydrolyze long-chain acyl-ACPs to produce free terminally branched LCFAs. Depending on the α-keto acid precursor, different terminally branched LCFA species can be formed [48]. For example, 3-methyl-oxobutyric acid can be converted to isobutyryl-CoA, which yields even-numbered iso-C14:0 and iso-C16:0 fatty acids. Precursors 4-methyl-oxopentanoic acid and 3-methyl-oxopentanoic acid will yield odd-numbered iso-branched and anteiso-branched C15:0 and C17:0 fatty acids, respectively [50].

### Regulation of terminally branched LCFA biosynthesis in Gram-positive bacteria

The plasma membrane of some Gram-positive bacteria contains a mixture of straight- and branched-chain lipids. Cells vary the proportion of branched-chain fatty acids to modulate membrane fluidity. Thus, regulating the biosynthesis of terminally branched LCFAs become important to cell fitness. In these bacteria, at least three layers of regulation of their LCFA composition are identified: (1) control the α-keto acid precursor pools, (2) selective expression of the *bkd* operon, and (3) control FabH substrate preferences (Fig. 2).

The *ilvB* operon involved in the biosynthesis of α-keto acids is regulated by CodY, a global transcriptional repressor [51]. Activation of CodY by guanosine triphosphate (GTP) and branched-chain amino acids allows CodY to bind to the promoter region of *ilvB*, leading to inhibition of α-keto acid biosynthesis [52]. In addition, the *ilvB* operon can also be transcriptionally activated by CcpA in response to glucose and repressed by TnrA in response to nitrogen [53].

The *B. subtilis bkd* operon is also regulated by several factors [54]. The promoter of the *bkd* operon is regulated by the sigma factor SigL, a member of the sigma 54 family. Transcription initiation from the *bkd* operon requires an activator protein BkdR, which interacts with an upstream activating sequence. The DNA binding activity of BkdR can be further enhanced by branched-chain amino acids as demonstrated in *Pseudomonas putida*, [55]. Additionally, cold shock can effectively stabilize *bkd* mRNAs, increasing the content of branched-chain fatty acids [56].

When exposed to cold temperatures, some Gram-positive bacteria predominantly increase the proportion of low-freezing anteiso-branched LCFAs relative to iso- and straight LCFAs in its membrane [50]. For example, the *Listeria monocytogenes* FabH showed a higher preference for 2-methylbutyryl-CoA, the precursor of odd-numbered anteiso-LCFAs, at 10 °C than that at 30 °C. The temperature-dependent substrate selectivity of FabH underlies the increased formation of anteiso-LCFAs during low-temperature adaptation [57].

### Metabolic engineering for the production of terminally branched long-chain fatty acids and their derivatives

**Fig. 3:**
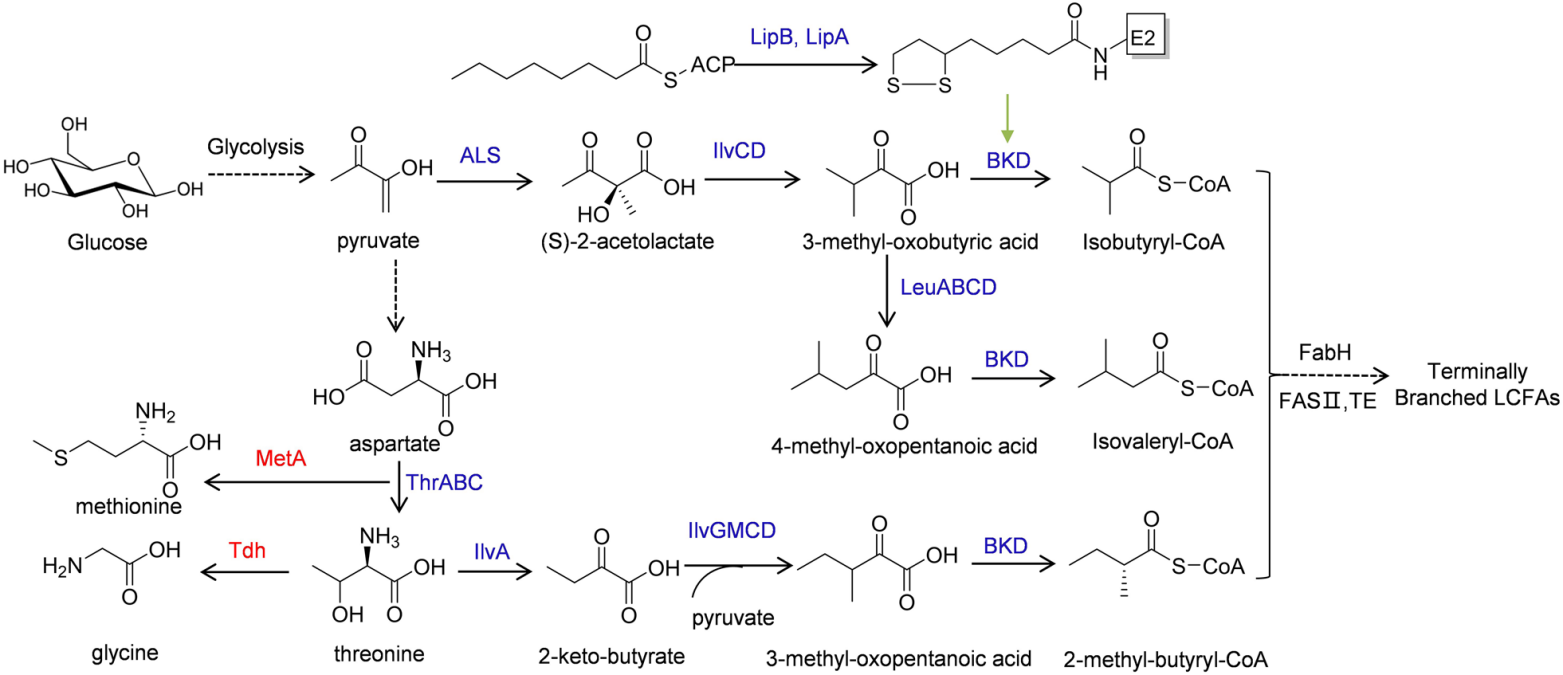
Engineering *E. coli* to produce terminally branched LCFAs directly from glucose. Overexpressed enzymes are shown by blue color; enzymes whose genes have been deleted from the chromosome are shown by red color. ALS: acetolactate synthase; BKD: branched-chain α-keto acid dehydrogenase; ThrA: aspartate kinase I/homoserine dehydrogenase I; ThrB: homoserine kinase; ThrC: threonine synthase; MetA: homoserine O-succinyltransferase; Tdh: threonine dehydrogenase; LipB: lipoyl transferase; LipA: lipoyl synthase

Recent advances in metabolic engineering have enabled the overproduction of terminally branched LCFAs in microbial hosts that naturally do not produce branched lipids. These engineering efforts have largely expanded the capability to bioproduce advanced biofuels in microbial hosts that have more attractive features than the native host [49]. By overexpressing the *B. subtilis fabH2* and its *bkd* operon, a terminally branched LCFA biosynthetic pathway was first constructed in *E. coli* (Fig. 3) [8]. However, the initially engineered strain only produced 2.5 mg/L of branched-chain LCFAs. Instead, a high proportion of straight-chain fatty acids were co-produced [8]. To increase the proportion of terminally branched LCFAs in total free fatty acids, Jiang et al. [58] replaced the acetyl-CoA-specific *E. coli* FabH with a branched-chain-acyl-CoA-specific FabH and found that this replacement directed the flux to the synthesis of terminally branched LCFAs and increased the terminally branched LCFAs titer by 48-fold. Later, it was found that overexpression of the *bkd* operon depleted the cellular lipoylation capability of the host, preventing the proper lipoylation of two *E. coli* native α-keto acid dehydrogenases, including the essential 2-oxoacid dehydrogenase (OADH) and the pyruvate dehydrogenase (PDH), thus inhibiting cell growth and terminally branched LCFAs production [59]. To solve this problem, an endogenous protein lipoylation pathway was engineered (Fig. 3). This pathway contains a lipoyl (octanoyl) transferase (LipB, encoded by *lipB*) that transfers an octanoyl moiety from octanoyl-ACP to the E2 subunit of α-keto acid dehydrogenases and a lipoyl synthase (LipA, encoded by *lipA*) that inserts two sulfur atoms into the octanoyl side chain of the octanoylated E2 subunit, forming a lipoyl group. The engineered protein lipoylation pathway not only restored the function of all α-keto acid dehydrogenases, but also increased the terminally branched LCFA titer to 207 mg/L [59]. Incorporation of the terminally branched LCFAs into the lipid membrane is expected to affect membrane fluidity and permeability, resulting in cellular stress. However, analysis of previously engineered LCFA-producing strains showed little incorporation of branched LCFAs into cell membrane [59].

While most Gram-positive bacteria produce both iso- and anteiso-LCFAs as a mixture, for practical applications, it is desirable to engineer strains that can specifically produce one type of terminally branched LCFAs. This has been achieved mostly by controlling the supply of α-keto acid precursors. When overexpressing the *B. subtilis alsS* and the *E. coli ilvCD* (Fig. 3), flux through 3-methyl-2-oxobutyric acid was enhanced, leading to even-chain-iso-fatty acid as the major terminally branched LCFA species (65%) [58]. When the *leuABCD* operon containing a feedback-resistant mutant of *leuA* was overexpressed, biosynthesis of 4-methyl-2-oxopentanoic acid was enhanced (Fig. 3), making the odd-chain-iso-fatty acids the predominant terminally branched LCFA products (89%) [58].

In addition, anteiso-branched LCFAs were produced as the major branched LCFA species by overexpressing the *Salmonella typhimurium ilvGMCD*, the *C. glutamicum ilvA*, and the *E. coli thrABC* to enhance the flux through 3-methyl-oxopentanoic acids and by dynamically regulating *fabH* expression (Fig. 3). The resulting strain produced anteiso-branched fatty acids up to 20.4% of total free fatty acids [60]. Overall, by engineering α-ketoacid biosynthetic pathways, compositions of terminally branched LCFAs can also be controlled.

Besides the overproduction of terminally branched LCFAs, some metabolic engineering works on the production of branched alkanes, alcohols, and esters have also been reported [7, 8]. Branched alkanes, as ideal biofuels, are structurally and chemically similar to fossil fuels. Howard et al. [8] introduced the fatty acid reductase complex from *Photorhabdus luminescens* and an aldehyde decarbonylase from *Nostoc punctiforme* into *E. coli* with a terminally branched LCFA biosynthetic pathway, yielding methyl pentadecane. The results clearly demonstrate the feasibility of engineering artificial pathways for branched alkane biosynthesis in a microbial host. Branched long-chain fatty alcohols (BLFLs) in the range of C12 to C18 are more suitable as diesel fuel replacements than their straight-chain counterparts. Jiang et al. [7] constructed and tested the efficiencies of four different biosynthetic pathways that convert branched acyl-ACPs to BLFLs in *E. coli*. A modular engineering approach was then used to balance the flux between α-keto acid synthesis, acyl-ACP generation, and alcohol formation. The best performing strain produced BLFLs at 350 mg/L, where 75% of the produced fatty alcohols were BLFLs. In addition, Tao et al. [61] combined the branched short-chain alcohol and the branched LCFA biosynthetic pathways in *E. coli*, and produced LCFA esters containing methyl branches at both acid and alcohol moieties at 35 mg/L with a yield of 1.9 mg/g glycerol.

## Internally branched fatty acid-derived fuels

Compared to straight and terminally branched LCFAs, internally branched LCFAs are relatively rare in nature. Thermophilic bacterium *Rubrobacter xylanophilus* produces 12-methylhexadecanoic acid and 14-methyloctadecanoic acid as the major fatty acid species in its lipids [62]. 13-Methylhexadecanoic acid was identified in *Leptogorgia piccolo* [63], and 9- and 12-methyltetradecanoic acids were found in cyanobacterium *Scytonema* sp. [64]. The best characterized internally branched LCFA is tuberculostearic acid (TSBA, 10-methylstearic acid) that is produced by *Mycobacterium tuberculosis* and related species [65]. TSBA is used as a clinical marker for the diagnosis of tuberculous and may play an important role in the persistent phase of infection [66]. Similarly, cyclopropane fatty acids (CFAs) that contain a cyclopropane ring represent another class of internally methyl-branched LCFAs. CFAs are major lipid components of many bacteria, such as *E. coli*, *S. typhimurium*, and *M. tuberculosis*. They are also found in the seed oils of some higher plants including *Malvales*, *Fabales*, and *Sapindales* [67]. The position of the methyl group has strong effects to physical properties of branched LCFAs and their derived biofuels, with the greatest effect occurring at the mid of the acyl chain [68]. For example, internally branched LCFAs have even lower freezing points compared with terminally branched LCFAs [69]. Therefore, internally branched LCFA-derived biofuels are more promising than those derived from terminally branched LCFAs. Until now, there has not been much engineering work toward the overproduction of internally branched LCFAs. Thus, we will focus on the recent developments on the understanding of their biosynthesis.

### Biosynthesis of internally branched LCFAs

**Fig. 4:**
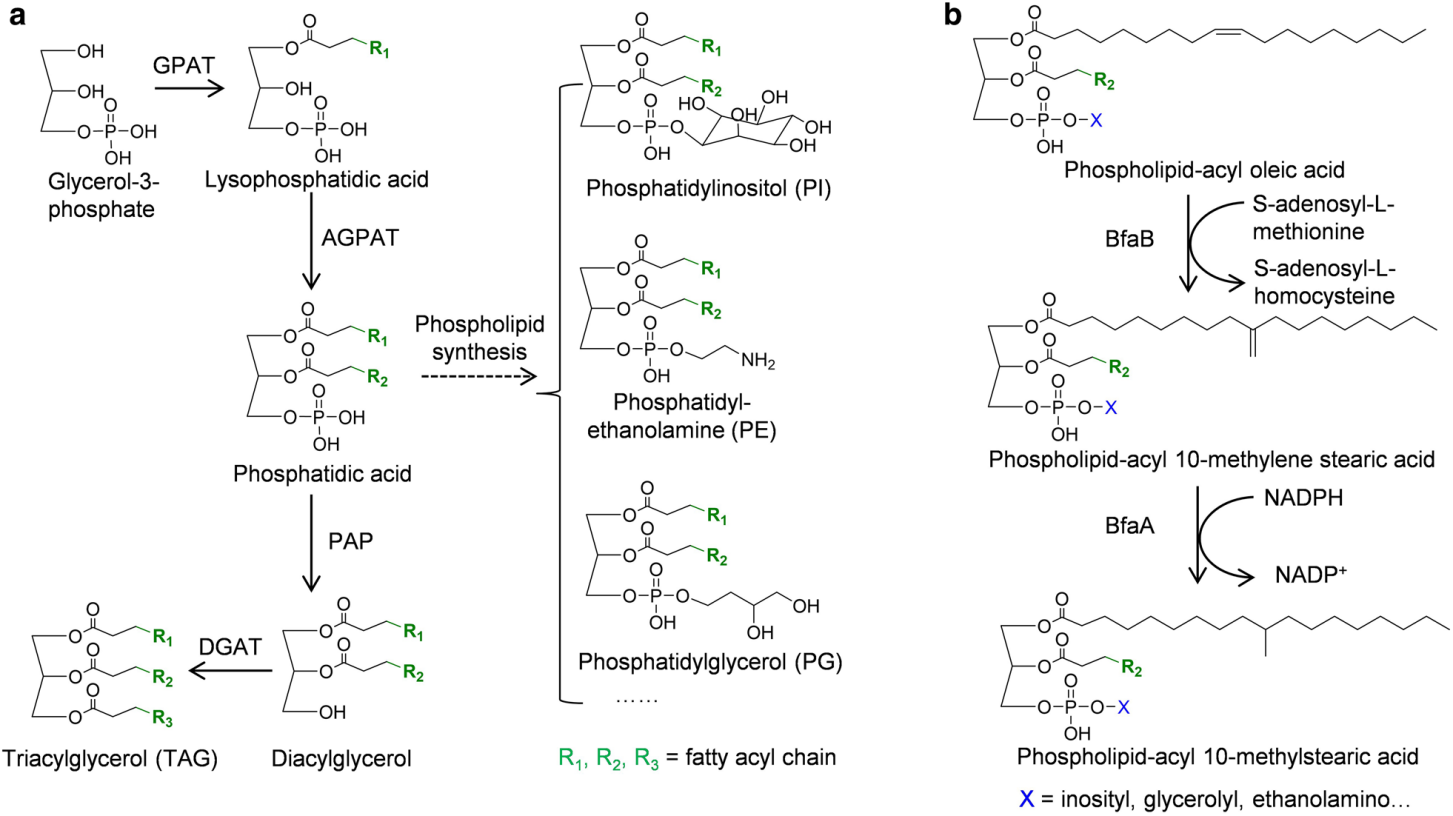
Phospholipid and tuberculostearic acid biosynthetic pathway. **a** The Kennedy pathway for triacylglycerol and phospholipid biosynthesis. GPAT: glycerol-3-phosphate acyl transferase; AGPAT: acylglycerol-3-phosphate acyl transferase; PAP: phosphatidic acid phosphatase; DGAT: diacylglycerol acyl transferase. **b** Proposed pathway for tuberculostearic acid biosynthesis. BfaB: S-adenosyl-l-methionine-dependent methyltransferase; BfaA: FAD-binding oxidoreductase

In *M. tuberculosis* and related species, TSBA has been found in multiple phospholipids such as phosphatidylethanolamine (PE), phosphatidylinositol (PI), and diphosphatidylglycerol (DPG), and glycolipids such as phosphatidylinositol mannosides and lipoarabinomannans [70]. These glycolipids are virulence determinants associated with *M. tuberculosis* and are likely involved in subverting the immune system [71]. Only small amounts of TBSA can be detected in purified triglycerides (TAGs) [72], indicating that the TSBA pathway enzymes recognize specific phospholipid substrates (Fig. 4a).

The mechanism for TSBA synthesis is still not fully understood. It was hypothesized that the biosynthesis of TSBA involves two reaction steps (Fig. 4b). The first step is the methylenation of the oleic acid (18:1Δ9) moiety of phospholipids by a methyltransferase using S-adenosyl-l-methionine (SAM) as the methyl donor. The second step is the reduction of the intermediates, which are believed to be 10-methylene-octadecanoyl phospholipids, by a reductase using NADPH as the cofactor [73]. Meena et al. [74] demonstrated that a methyltransferase encoded by *umaA* from *M. tuberculosis* H37Rv is capable of converting the oleoyl-phosphatidylcholine (PC) to TSBA-PC in vitro. Meena et al. [75] also showed that another methyltransferase encoded by *ufaA1* can also catalyze the formation of TSBA. Purified recombinant UfaA1 protein can convert oleoyl-phosphatidylcholine and oleoyl-PE to TSBA-PC and TSBA-PE in the presence of SAM and NADPH. However, neither *umaA* nor *ufaA1* has any functional domain associated with redox reactions. Machida et al. [45] later identified a gene cluster from *Mycobacterium chlorophenolicum* that is responsible for TSBA production. In this cluster, the *bfaB* gene encodes a SAM-dependent methyltransferase, and the *bfaA* gene encodes a FAD-dependent oxidoreductase. Heterologous expression of these two genes in *E. coli* produced TSBA from oleic acid, confirming their functions. These two enzymes, BfaA and BfaB, were found to have surprisingly high regioselectivity, only converting 18:1Δ9, but not 16:1Δ9 nor 18:1Δ11 to branched fatty acids when tested in *E. coli*. Furthermore, it is not clear which phospholipids can be the substrates of the TSBA enzymes. Additionally, because unsaturated fatty acids (UFAs) are usually located at the *sn*-2 position of phospholipids in *E. coli*, TSBAs produced in *E. coli* are likely located at *sn*-2 position [76]. Meanwhile, in mycobacteria, TSBA is usually found at the *sn*-1 position of phospholipids. These results suggest that BfaA and BfaB might be able to convert UFAs at both *sn*-1 and *sn*-2 positions of phospholipids [77, 78]. Further studies are needed to illuminate their substrate specificities.

### Biosynthesis of CFAs

Similar to other branched LCFAs, CFAs are also believed to modulate the fluidity and stability of cell membranes. CFAs are important to improve cell survival when microbes are subjected to environmental stresses such as high osmotic pressure [79], high (low) temperature [79, 80], low pH [81], and organic solvent conditions [82]. Specifically, an increased level of CFAs was observed in *E. coli* after exposure to pH 4 for 16 h [83]. Deletion of CFA biosynthetic genes in *P. putida* results in decreased cell tolerance to organic solvents [84]. Additionally, CFAs also play an important role in bacterial virulence and persistence. For example, deletion of the *M. tuberculosis pcaA* gene, which encodes a CFA synthase and acts on α-mycolates, inhibits *M. tuberculosis* from killing infected mice [85].

**Fig. 5:**
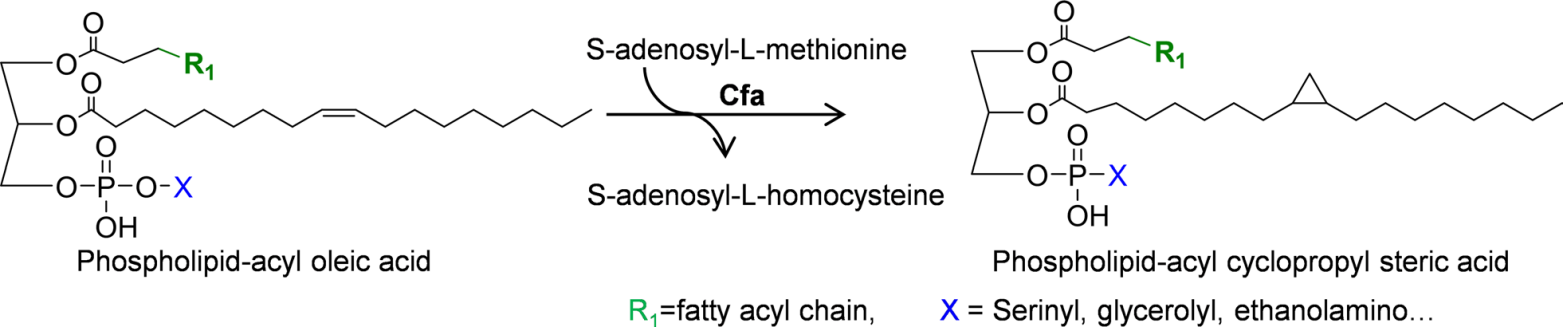
Pathway for cyclopropane fatty acid biosynthesis. Cfa: cyclopropane fatty acid synthase

Biosynthesis of CFAs is catalyzed by cyclopropane fatty acid synthase (Cfa), which transfers a methylene group from SAM to the double bond of UFAs, creating a cyclopropane ring on the alkyl chain (Fig. 5). CFAs are typically produced from preexisting *cis*-UFAs of phospholipids through the Kennedy pathway (Fig. 4a). Therefore, most natural CFAs retain the *cis* configuration [86]. An in vitro study of the *E. coli* Cfa indicates that this enzyme has activities on different types of phospholipids including PE, phosphatidylglycerol (PG), cardiolipin (CL), and PC. In addition, the *E. coli* Cfa was reported to prefer preferentially act the *sn*-2 position of phospholipids [87], whereas Cfa from *Sterculia foetida* acts on *sn*-1 position [88].

The formation of CFAs occurs at the onset of the stationary phase in the cell growth cycle and continues until all of the *cis*-UFAs of the membrane phospholipid bilayers are converted into CFAs. This growth phase-depended regulation of CFA synthesis is caused by a RpoS-dependent promoter, which is activated only when cells enter stationary phase. In addition, the activity of the Cfa protein is also believed to be controlled by an unidentified energy-independent protease that is transcribed by a RpoH-dependent promoter [89].

Although CFAs has not been currently used as biofuels, their lower melting temperature compared to straight LCFAs and improved stability against oxidation compared to unsaturated FAs have made them promising biofuel targets as recently explored [90]. By introducing the *cfa* gene from *E. coli* into cyanobacterium *Synechocystis* sp. PCC 6803, CFAs were produced in engineered cells with up to 30% of total fatty acids [90]. In addition, engineering their biosynthesis could improve the tolerance of bacterial host to harsh conditions. For example, overexpression of *cfa* in *C. acetobutylicum* ATCC 824 showed an increased butanol tolerance [91], while a *cfa*-deficient *E. coli* [92] or *S. typhimurium* [93] was very sensitive to acid stress. Yu et al. [94] expressed the *E. coli cfa* gene in the seeds of *Arabidopsis* and resulted the accumulation of CFAs up to 9.1%. By coexpressing a *Sterculia foetida* lysophosphatidic acid acyltransferase (SfLPAT), the CFA content was further increased to 35%.

## Conclusions

We reviewed the progress of branched-chain biofuels, including branched short- and long-chain alcohols, alkanes, and esters. As we understand more about the biochemistry and regulation of lipid biosynthesis, novel metabolic pathways have been continuously developed, leading to new generations of biofuels with structures and properties highly similar to or identical with existing petroleum-derived fuels. The reviewed studies have proved the concept of producing advanced biofuels from engineered microorganisms; however, current titers and yields are still too low for economically viable production. These challenges demand efficient metabolic engineering tools to improve pathway yields, titers, and production stability of engineered microbial strains. Fortunately, some tools are already emerging and used for biofuel production, for example, modeling-guided pathway optimization [95], metabolite biosensor-enabled high-throughput screening and dynamic pathway regulation [96], and microbial population control tools to improve ensemble production [97]. Additionally, there is a clear trend to move these pathways and engineering strategies into industry-relevant microbial hosts. Hopefully, these current and future efforts will enable the conversion of various low-cost, abundant and environmental-friendly feedstock into advanced branched-chain fuels at high titers, yields, and productivities in industry-relevant scales.

## Abbreviations

ACP: acyl carrier protein
ADH: alcohol dehydrogenases
AGPAT: acylglycerol-3-phosphate acyl transferase
ALDH: aldehyde dehydrogenase
ALR: aldehyde reductase
ALS: acetolactate synthase
ATF: alcohol acetyltransferases
BKD: branched-chain α-keto acid dehydrogenases
BLFL: branched long-chain fatty alcohol
CFA: cyclopropane fatty acids
CL: cardiolipin
DGAT: diacylglycerol acyl transferase
DPG: diphosphatidylglycerol
FabH: β-ketoacyl-acyl carrier protein synthase III
FASII: type II fatty acid biosynthesis system
GPAT: glycerol-3-phosphate acyl transferase
KDC: α-ketoacid decarboxylase
LCFAs: long-chain fatty acids
OADH: 2-oxoacid dehydrogenase
PDC: pyruvate decarboxylase
PDH: pyruvate dehydrogenase
PC: phosphatidylcholine
PE: phosphatidyl ethanolamine
PG: phosphatidylglycerol
PI: phosphatidylinositol
PAP: phosphatidic acid phosphatase
SAM: S-adenosyl-l-methionine
TE: thioesterase
UFA: unsaturated fatty acid
